# PLD3 is a neuronal lysosomal phospholipase D associated with β-amyloid plaques and cognitive function in Alzheimer’s disease

**DOI:** 10.1371/journal.pgen.1009406

**Published:** 2021-04-08

**Authors:** Alex G. Nackenoff, Timothy J. Hohman, Sarah M. Neuner, Carolyn S. Akers, Nicole C. Weitzel, Alena Shostak, Shawn M. Ferguson, Bret Mobley, David A. Bennett, Julie A. Schneider, Angela L. Jefferson, Catherine C. Kaczorowski, Matthew S. Schrag

**Affiliations:** 1 Department of Neurology, Vanderbilt University Medical Center, Nashville, Tennessee, United States of America; 2 Vanderbilt Memory and Alzheimer’s Center, Department of Neurology, Vanderbilt University Medical Center, Nashville, Tennessee, United States of America; 3 The Jackson Laboratory, Bar Harbor, Maine, United States of America; 4 Department of Cell Biology, Yale University, New Haven, Connecticut, United States of America; 5 Department of Pathology, Vanderbilt University Medical Center, Nashville, Tennessee, United States of America; 6 Rush Alzheimer’s Disease Center, Rush University Medical Center, Chicago, Illinois, United States of America; HudsonAlpha Institute for Biotechnology, UNITED STATES

## Abstract

Phospholipase D3 (PLD3) is a protein of unclear function that structurally resembles other members of the phospholipase D superfamily. A coding variant in this gene confers increased risk for the development of Alzheimer’s disease (AD), although the magnitude of this effect has been controversial. Because of the potential significance of this obscure protein, we undertook a study to observe its distribution in normal human brain and AD-affected brain, determine whether PLD3 is relevant to memory and cognition in sporadic AD, and to evaluate its molecular function. In human neuropathological samples, PLD3 was primarily found within neurons and colocalized with lysosome markers (LAMP2, progranulin, and cathepsins D and B). This colocalization was also present in AD brain with prominent enrichment on lysosomal accumulations within dystrophic neurites surrounding β-amyloid plaques. This pattern of protein distribution was conserved in mouse brain in wild type and the 5xFAD mouse model of cerebral β-amyloidosis. We discovered PLD3 has phospholipase D activity in lysosomes. A coding variant in PLD3 reported to confer AD risk significantly reduced enzymatic activity compared to wild-type PLD3. PLD3 mRNA levels in the human pre-frontal cortex inversely correlated with β-amyloid pathology severity and rate of cognitive decline in 531 participants enrolled in the Religious Orders Study and Rush Memory and Aging Project. PLD3 levels across genetically diverse BXD mouse strains and strains crossed with 5xFAD mice correlated strongly with learning and memory performance in a fear conditioning task. In summary, this study identified a new functional mammalian phospholipase D isoform which is lysosomal and closely associated with both β-amyloid pathology and cognition.

## Author summary

A rare variant in phosphlipase D3 (PLD3) was linked to Alzheimer’s disease (AD) risk, but both the impact of the variant on protein’s function and the role of PLD3 in sporadic AD are unknown. Here we show that PLD3 is a lysosomal phospholipase D and that the AD-associated variant impairs its function. We also discovered that PLD3 expression levels correlate β-amyloid plaque density and the rate of cognitive decline in the longitudinal Religious Orders Study and Rush Memory and Aging Project. Finally, we observed PLD3 expression levels correlated with memory and learning in a genetically diverse mouse model. These discoveries establish that PLD3 is an important AD risk gene and implicate PLD3 in the neurobiology of lysosomal dysfunction in AD.

## Introduction

Alzheimer’s disease (AD) is the most common form of dementia and an increasing societal and economic burden. Multiple gene variants were recently discovered in the Phospholipase D3 (PLD3) gene which conferred increased risk for late onset AD [1]. Phospholipase D enzymes (PLDs) form a superfamily that are present in viruses, bacteria, and plants all the way up to mammalian cells. PLDs play an important role in the conversion primarily of phosphatidylcholine to phosphatidic acid and choline, which has implications for membrane dynamics and cell signaling, among other important cellular processes [2]. The phosphodiesterase domains of PLDs contain two highly conserved catalytic regions, HxKxxxxD motifs (hereafter HKD motif). There are a few non-canonical functions of PLDs which include nuclease activity and cardiolipase activity, both of which are mediated by substrate binding at the HKD motif [3,4]. Phospholipase D1 and D2 (PLD1 and PLD2), are confirmed to be enzymatically active mammalian PLD isoforms and are druggable proteins [2,5]. PLD3 was identified as a PLD based upon homology as it contains two HKD motifs, but two prior studies informally reported that PLD3 lacks PLD activity, although the data and methodology were not presented [6,7]. The PLD3 gene variants associated with increased late onset AD risk were initially linked to β-amyloid precursor protein (APP) processing [1] but this could only be replicated in overexpression conditions [8]. Moreover, a number of subsequent reports failed to replicate the association of the highest LOAD risk PLD3-V232M coding variant, although this may have been due in part to the rarity of the coding variant [9–12]. In a later meta-analysis, the PLD3-V232M contributed to AD risk but had a smaller effect than initially reported, comparable in magnitude to the apolipoprotein E-ε4 allele [13]. The V232M variant is associated with decreased PLD3 expression and is adjacent to an HKD motif, suggesting PLD3 hypofunction may drive or modulate AD pathology. Because the molecular function of this protein in the brain is unclear, it is not known whether this variant affects protein function. Additionally, because the variant is rare (~1% of affected patients)[1], the relevance of PLD3 to AD processes at a population level is yet unknown.

In all, there has been little resolution to the controversy whether PLD3 is a legitimate AD-risk gene, in part due to the limitations of studying rare coding gene variants. In this study, we aim to address the impact of PLD3 upon AD-related biological and cognitive pathologies leveraging generalizable population level datasets as well as determining the molecular function of PLD3.

## Methods

### Ethics statement

Tissue procurement was approved by the Vanderbilt University Medical Center Institutional Review Board and all specimens were de-identified. Written consent for brain donation was obtained from patients or their surrogate decision makers. Ethical oversight and approval of animal use was provided by Vanderbilt University Medical Center’s Institutional Animal Care and Use Committee.

### Immunohistochemistry

#### Human AD brain samples

Formalin fixed brain tissue from sixteen human participants was obtained from autopsy, including eight cases of autopsy-verified Alzheimer’s disease and eight neurological controls (**Table A in S1 Text**). Post-mortem interval was less than 48 hours for all cases.

#### Mouse brain samples

WT and 5xFAD 3-month-old mice were euthanized following ketamine/xylazine anesthesia and transcardial perfusion with ice cold PBS. One hemisphere of each mouse was used for immunohistochemistry and was drop fixed in ice-cold 4% paraformaldehyde/PBS. Brains were left to fix for at least one week at 4°C before slicing.

#### Brain sectioning & staining

Fixed brain tissue was sectioned at 50 μM on a Leica vibratome. Sections were then subjected to antigen retrieval, consisting of firstly heating to 95°C in citric acid buffer (10 mM citric acid, 0.05% Tween-20, pH 6) for 20 min. Slices were then transferred to PBS/glycine buffer (100 mM glycine/1x PBS, 0.1% Tween-20) for 10 min shaking at room temperature, then washed in PBS/0.1% Triton X-100 for 10 min at room temperature. Sections were then placed in blocking buffer (4% Bovine serum albumin (BSA) in PBS/0.1% Triton X-100) under a 240-watt LED panel with emission at 390 nm, 430 nm, 460 nm, 630 nm, 660 nm and 850 nm (GrowLight, HTG Supply Inc, USA) for a minimum of 18 hours at 4°C to reduce autofluorescence related to lipofuscin. Primary antibodies were applied in 4% BSA in PBS/0.1% Triton X-100 overnight at 4° C with gentle shaking (see **Table B in S1 Text**). Slices were then washed 3 x 10 min room temperature in PBS/T before application of fluorescent secondary antibody in 4% BSA/PBS/T for a minimum of 2 hours room temperature incubation. Slices were finally washed 3 x 10 min room temperature in PBS/T before slide mounting and coverslipping.

#### Microscopy

Tissue was visualized on a laser scanning confocal microscope (Zeiss, LSM 710) with a 20x objective or 63x oil immersion objective. Images were acquired with a minimum resolution of at least 1024x1024 pixels. All images underwent routine processing with the Image J software (https://imagej.nih.gov/ij/download.html). Colocalization was assessed with Pearson’s correlation coefficient and analyzed with MIPAV image analysis software (https://mipav.cit.nih.gov/download.php).

### AD-BXD studies

#### AD-BXD mouse line

Mice were generated as previously described [14,15]. Briefly, stable inbred BXD mouse lines (generating an array of genetic diversity between C57BL/6 and DBA2/J) were crossed with 5xFAD hemizygous mice, which contain five induced variants known to cause familial AD in human [16]. Due to the hemizygous nature of 5xFAD, 50% of the F1 progeny carry the 5xFAD insert (AD-BXD) and 50% are WT for littermate controls (WT-BXD), where all mice contain the respective BXD line gene diversity. Experiments were performed at 6 months and 14 months of age upon both male and female mice. Mice underwent contextual fear conditioning (n = 636), of which a subset was euthanized and brain tissue subjected to RNA-Seq (n = 133). Dots represent averaged behavioral performance within BXD background strain and PLD3 transcript count (n = 100 strains).

#### Contextual fear conditioning

Following three days of habituation to transport and to the testing environment, mice were trained on a standard contextual fear conditioning paradigm as previously described [14,15]. Contextual fear training consisted of a 180 s baseline period followed by four mild foot shocks (1 s, 0.9 mA), separated by 115±20 s. A 40 s interval following each foot shock was defined as the post-shock interval, and the percentage of time spent freezing during each of these intervals was measured using FreezeFrame software (Coulbourn Instruments, PA, USA). To calculate an acquisition curve for each strain as an index of learning, the slope across the average time spent freezing during post-shock intervals 1–4 was derived. Twenty-four hours later, hippocampus-dependent contextual fear memory (CFM) was tested by returning the mouse to the testing chamber for 10 min. The percentage of time spent freezing during the testing trial was measured using FreezeFrame software and used as an index of CFM.

#### RNA-Seq

RNA was quantified via RNA-Seq as previously described [14], and available on GEO (https://www.ncbi.nlm.nih.gov/geo/query/acc.cgi?acc=GSE101144). Briefly, snap frozen hippocampi from AD-BXD strains and non-carrier littermate controls at 6 and 14 months were used for RNA sequencing. RNA was isolated using the RNeasy mini kit (Qiagen) and treated with DNase to remove contaminating DNA. Final library pools were sequenced by 75bp paired-end sequencing on a HiSeq2500 (Illumina, San Diego, CA, USA). For final by-strain analysis, samples belonging to the same strain/sex/age/genotype group were averaged.

### ROS-MAP cohort and analysis

Data for human analyses were acquired from two cohort studies acquired and made available by the Rush University AD center (RADC), all data of which are freely available through the RADC web portal (www.radc.rush.edu). The Religious Orders Study (ROS) began in 1994 and the Rush Memory and Aging Project (MAP) began in 1997. Both studies enrolled older adults without dementia and all participants signed an Anatomical Gift act for brain donation [17–19]. Written informed consent was obtained from all participants and all protocols were approved by the Institutional Review Board (IRB). Secondary analyses of data were approved by the Vanderbilt University Medical Center Institutional Review Board.

#### Clinical diagnosis

As described in detail previously [20], all clinical and neuropsychological data were reviewed by a board-certified neurologist at the time of death and a summary diagnostic opinion was made blinded to all postmortem data. Consensus diagnosis was reached in case conference for selected cases.

#### RNA sequencing

RNA expression levels were obtained, quantified, processed, and normalized previously, and have been described in detail elsewhere [21,22]. Briefly, RNA expression levels were obtained from frozen, manually dissected sections of dorsolateral prefrontal cortex. RNA was isolated using the RNeasy lipid tissue kit (Qiagen, Valencia, CA) and was reverse transcribed and biotin-UTP labeled using the llumina TotalPrep RNA Amplification Kit from Ambion (Illumina, San Diego, CA, USA). Expression signals were generated using BeadStudio (Illumina, San Diego, CA, USA). Standard control and normalization methods were employed to account for technical variability due to differences in hybridization dates. Reads were aligned using the Bowtie 1 package and then counted using RSEM, as previously described [22].

#### Neuropathology

Measures of AD neuropathology were previously quantified and described in detail elsewhere [17,18,22]. β-amyloid and tau burden were quantified in eight brain regions using immunohistochemistry including the superior frontal cortex, anterior cingulate, hippocampus, angular gyrus, entorhinal cortex, calcarine cortex, middle frontal cortex, and inferior temporal cortex. Neuritic plaques and neurofibrillary tangles were quantified from silver stained slides, including five brain regions (middle frontal cortex, middle temporal cortex, inferior parietal cortex, hippocampus, and entorhinal cortex). Each AD neuropathology outcome was square-root transformed to better approximate a normal distribution.

In addition, non-AD pathologies were previously quantified and described in detail elsewhere [23–29] including TDP-43[30], cerebral amyloid angiopathy (CAA)[25], microscopic infarcts [24], gross infarcts [28], arteriolosclerosis [26], and atherosclerosis [23]. The quantification and modeling of each of these outcomes for the present analytical models has been described previously [22].

#### Cognitive outcomes

Cognitive performance was evaluated with 19 neuropsychological tests as previously described [31]. Tests probed multiple cognitive domains, including semantic memory, working memory, episodic memory, perceptual speed, and perceptual organization. Z-scores from all tests were averaged into a single global cognitive composite as previously described [31].

#### Statistical analyses

All statistical analyses were performed in R (version 3.5.0; https://www.r-project.org/). Differential expression of *PLD3* across diagnostic groups was assessed using linear regression with *PLD3* entered as a continuous outcome and diagnosis as a categorical predictor with the normal cognition group set as the referent. Linear regression models assessed the association between *PLD3* and neuropathological burden at autopsy with square-root transformed AD neuropathology traits set as continuous outcomes in separate models. In all models, covariates included age at death, sex, and postmortem interval.

For cognitive analyses, linear regression models assessed the association between *PLD3* expression and cognitive performance at last visit prior to death in the same manner outlined above. Longitudinal change in cognition was evaluated using mixed-effects regression models with the intercept and interval (years from death) entered as both fixed and random effects and included the same covariates as the cross-sectional models. PLD3 associations with longitudinal change were assessed with a *PLD3* x interval interaction term in the model.

### Cell culture and PLD3 expression

HeLa and NSC34 cells were cultured at 37° C and 5% CO_2_ in DMEM cell media containing 10% fetal bovine serum and penicillin and streptomycin. Cells were plated onto 150 mm culture flasks and transfected with 20 μg of pCMV3-hPLD3 (HG16349-NH; Sino Biological, Wayne, PA, USA) utilizing Fugene 6 (Promega, USA) at ~75% confluency. Plasmids containing hPLD3-V232M and K418R mutations were created using site-directed mutagenesis against PLD3 (E0552S, New England BioLabs). The sequence of all plasmids was confirmed prior to use. siRNA against PLD3 (159175867; Integrated DNA Technologies, Coralville, Iowa USA) was transfected using Oligofectamine (Invitrogen, USA) and control conditions were transfected with a scrambled siRNA. Target specificity of siRNA and efficiency were confirmed by western blot (**Fig A in S1 Text**) and we confirmed that reduction of PLD3 did not alter the level of PLD1 or PLD2 on lysosomes (**Fig B in S1 Text**). Experiments were performed 48 h after transfection.

### Lysosomal isolation and enzymatic assay

Lysosomes were isolated similarly to procedures previously described [32]. Briefly, cells were grown to confluence on a 150 mm dish and treated for 2 hours with a colloid suspension of dextran-coated iron oxide nanoparticles at a concentration of 10mg/mL in DMEM containing 10% fetal bovine serum and penicillin and streptomycin. The cells were then washed three times and allowed to rest for at least 2 hours prior to collection. Cells were scraped free in 1 mL cold homogenization buffer consisting of PBS containing a protease inhibitor cocktail. The material was homogenized with 8 strokes of a tight fitting Dounce homogenizer and was applied to a column containing 50 mg fine steel filaments which was placed on a strong magnet. The cellular material passing through the column was collected and applied to the column two more times. The column was then washed with 10 mL cold homogenization buffer. The column was then placed in a microcentrifuge tube and centrifuged at 10,000 g for 10 minutes yielding the lysosomal fraction. Approximately 1.5–3% of total protein was recovered in this fraction. The isolation procedure was conducted in less than 1 hour and all steps were performed on ice. Protein concentration was assessed via the Bradford method (Bio-Safe #1610786, Bio-Rad, USA). Equal protein of intact lysosomes was loaded in replicate onto 96-well plates and then incubated with Amplex Red PLD assay kit, per manufacturer instructions (#A12219, ThermoFisher Scientific, USA). Cabbage-derived PLD was used as a positive control (#P8398, Sigma-Aldrich). Samples were incubated at 37° C for 1 hour, and read on a fluorescent plate reader (POLARstar Omega; BMG Labtech, USA) at excitation: 571 nm/emission: 585 nm.

### Western blot

Cells were scraped from 150 mm dishes in 1 mL cold PBS containing 1% Triton X-100 and a protease inhibitor cocktail. Tissue was homogenized in the same buffer using a 10 strokes of a Dounce homogenizer. Lysates were rotated end-over-end at 4° C for 60 minutes, then centrifuged at 10,000 g for 10 minutes to remove crude cellular debris. Protein concentration of the resulting lysate was determined with a Bradford assay. The lysate was combined with an equal volume of 2x Laemmelli’s buffer containing beta-mercaptoethanol and heated to 95° C for 10 minutes to denature. Equal protein mass was loaded into each lane of a pre-cast 4–15% gradient acrylamide gel and run at 200 V. Transfer was performed in a semi-dry apparatus (Trans-Turbo, Bio-Rad, USA). PVDF membrane were then blocked in 4% bovine serum albumin (BSA) in phosphate buffered saline (PBS) containing 0.1% Tween-20 (PBST) for 60 minutes. They were then incubated with primary antibodies diluted in 4% BSA in PBST overnight. Membranes were then washed 3 x 10 min in PBST prior to incubation with secondary antibody (diluted in 4% BSA in PBST) for 2 hours. Membranes were washed three times again, treated with Clarity chemiluminescence reagent (#1705061, Bio-Rad, USA) and developed on a chemiluminescence imaging system (12003153, ChemiDoc Imaging System, Bio-Rad, USA)

#### Graphical and statistical methods

All data were graphed and statistically analyzed using Prism 6.0 (GraphPad, Inc., La Jolla, CA) or R, as noted. In all statistical tests, P value significance thresholds were set *a priori* to α = 0.05. Specific statistical tests to assess main effects and post-hoc tests as well as N for all groups are provided in the figure legends.

## Results

### PLD3 is a lysosomal protein and enriched around β-amyloid plaques

**Fig 1:**
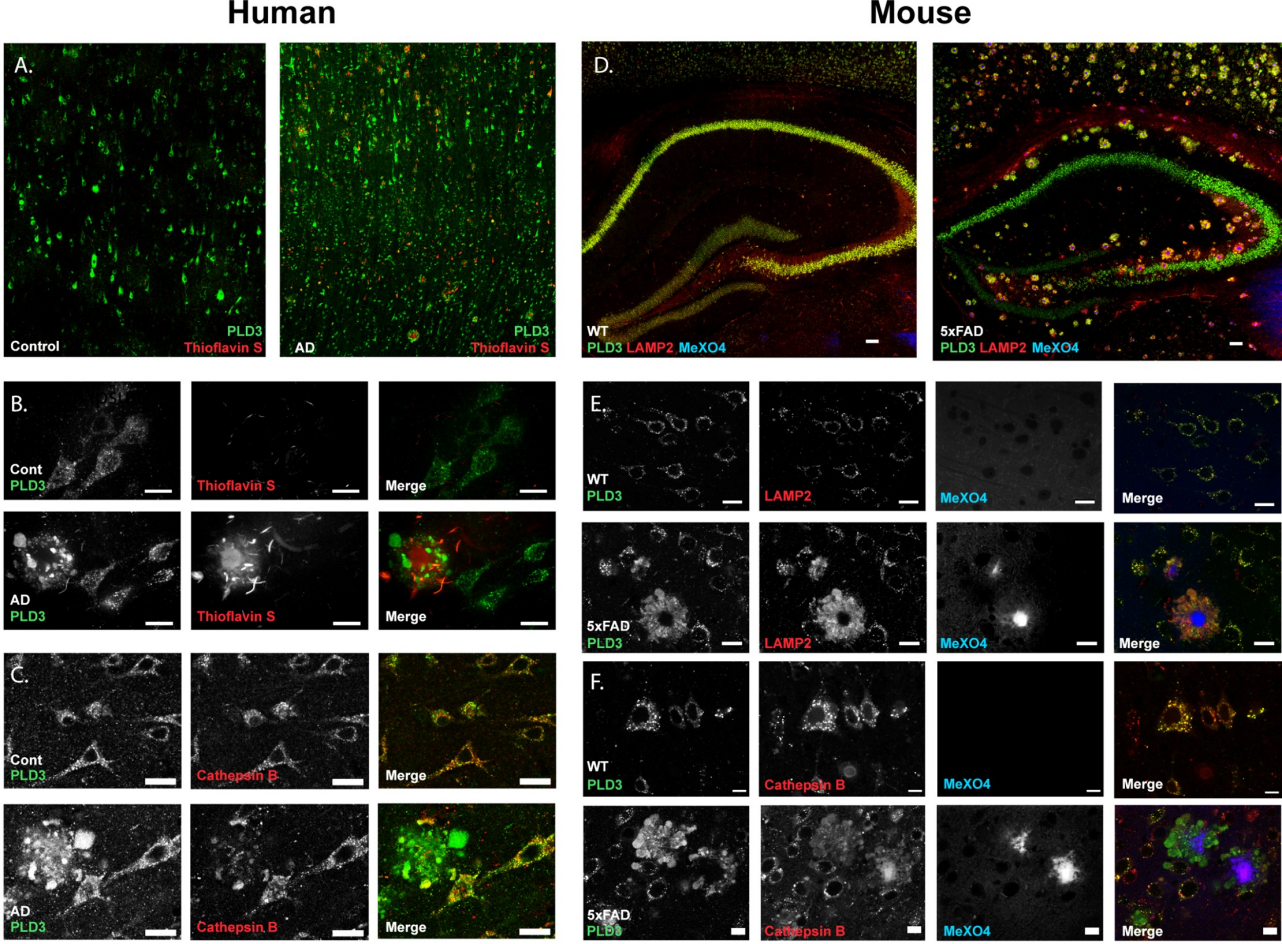
PLD3 is enriched in lysosomes surrounding parenchymal β-amyloid plaques in AD tissue. **A.** Low magnification images of temporal lobe cortex from neurological control and AD brain demonstrated a neuronal pattern of staining against PLD3 (green) and consistent accumulation around β-amyloid plaques. **B.** 40x magnification images of the same immunohistochemistry show PLD3 is in a punctate staining pattern within neuronal cell bodies and enriched around β-amyloid plaques. **C.** Co-staining with lysosomal marker cathepsin B confirmed PLD3 was primarily lysosomal and enriched in dystrophic neurites in AD brain. **D.** In 5xFAD mice, PLD3 was similarly enriched around every β-amyloid plaque and strongly colocalized with LAMP2. **E.** 40x magnification of WT and 5xFAD tissue demonstrated strong co-localization of PLD3 with lysosomal membrane marker LAMP2 (Pearson coefficient: 0.84±0.07 for WT, 0.86±0.04 for 5xFAD) and strong staining in dystrophic neurites around β-amyloid plaques, which are stained blue with methoxy-X04. **F.** The lysosomal lumenal protease cathepsin B is similarly co-localized with PLD3 in both normal and diseased neurons.

**Fig 2:**
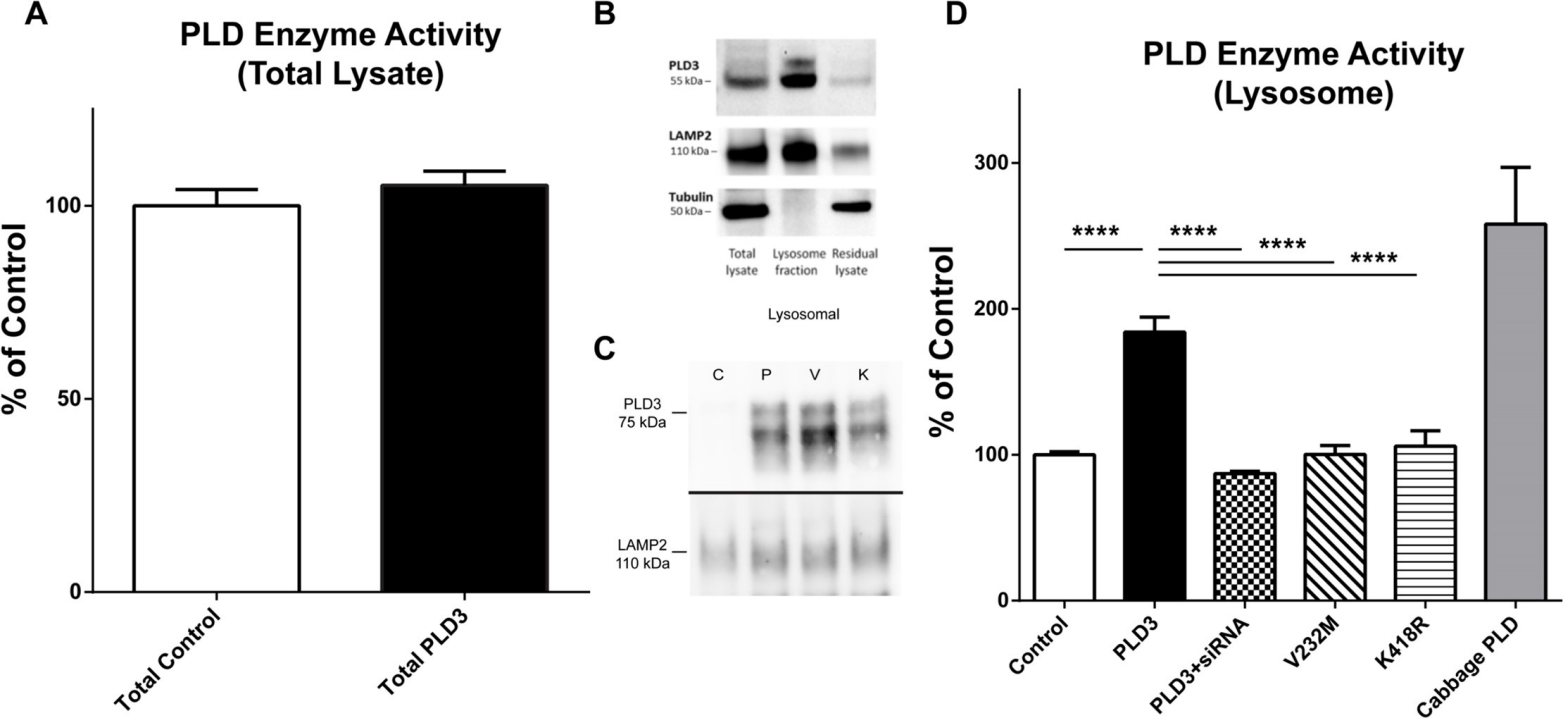
PLD3 has phospholipase D activity in isolated lysosomes. **A.** Overexpression of PLD3 does not produce increased phospholipase D activity in total lysate of NSC cells compared to untransfected NSC total lysate. N = 4 biological/assay replicates. **B.** Lysosomes isolated following transfection of PLD3 via iron dextran nanoparticles and magnetic affinity columns display enrichment for lysosomal marker LAMP2 and expected lack of cytoskeletal marker tubulin. Lysosomal fraction is enriched with PLD3, further indicating that PLD3 is a lysosomal protein. **C.** Lysosomal fractionation demonstrates that overexpressed wild-type PLD3 (*P*) is appropriately trafficked to the lysosomal fraction. Similarly, introduction of the AD-associated PLD3 mutation V232M (*V*) or a mutation in the HKD domain, K418R (*K*), did not impact routing to the lysosome (representative blot shown), indicated by Western Blot. Samples isolated via lysosomal isolation detailed in the Methods section. **D**. Data derived from lysosomes isolated from transfected NSC cells, each condition containing n = 4 biological/assay replicates per condition and n>16 sample replicates per condition. Phospholipase D (PLD) activity measured by the Amplex Red PLD assay with negative (control lysosomes) and positive (cabbage PLD) controls in each assay run. Lysosomes expressing PLD3 display significant increase in PLD activity compared to control lysosomes, which is similar in magnitude to the PLD activity seen with purified cabbage PLD. Upon cotransfection of siRNA against PLD3 alongside PLD3 transfection, these lysosomes display no PLD activity above the control condition. PLD3-K418R transfected lysosomes, which contain a mutated lysine residue in its putative HKD domain, have significantly reduced PLD activity compared to wild-type PLD3 transfected lysosomes. Furthermore, human AD-risk associated variant PLD3-V232M displays no significant PLD activity, and displays a significant reduction in PLD activity compared to PLD3. Data represented as mean +/- SEM. Data analyzed via one-way ANOVA. ****p<0.0001 following Bonferroni post-hoc tests.

While most early reports suggested PLD3 was a neuronal endoplasmic reticulum-localized protein [33], a few studies suggested it may, at least partially, localize to the lysosome. Most of these claims were based on studies of overexpression of the protein [34,35]. Because of contradictory reports regarding the subcellular localization of PLD3, we sought to better resolve the subcellular localization of PLD3 with attention to endogenous context-relevant tissue. In human brain tissue, PLD3 staining in neuronal cell bodies colocalized highly with Cathepsin B (**Fig 1**) (mean Pearson correlation coefficient 0.84±0.07 for neurological controls, 0.86±0.04 for AD) and other lysosomal markers LAMP2 and Cathepsin D (**Fig C in S1 Text**). In AD, β-amyloid plaques are surrounded by enlarged axon segments, termed dystrophic neurites, which are filled with lysosome-like organelles [35] (**Fig 1**). PLD3 was enriched and strongly localized with lysosomal membrane markers in dystrophic neurites around β-amyloid plaques (**Fig 1**), although the association with lumenal markers Cathepsin B/D was attenuated compared to cell bodies (**Fig 1**), consistent with a prior report that lysosomes in dystrophic neurites are deficient in luminal proteases [36]. These data confirm that even around β-amyloid plaques, PLD3 remains on lysosomes. We found minimal association of PLD3 with arteriolar CAA; however, dystrophic neurites form around capillaries with CAA and PLD3 was enriched in these neurites (**Fig D in S1 Text**). Similarly, in brain tissue from wild-type and 5xFAD mice PLD3 was strongly colocalized with markers of lysosomes (Pearson coefficient: 0.72±0.22 for WT, 0.65±0.10 for 5xFAD). The AD mouse model 5xFAD recapitulates this phenotype (**Fig 1**). Furthermore, in 5xFAD PLD3 did not colocalize with β-amyloid (Pearson correlation coefficient 0.13±0.10), but was enriched on lysosome-like organelles within dystrophic neurites surrounding β-amyloid plaques. Finally, to confirm that PLD3 is a lysosomal protein, lysosomes were isolated from non-transfected HeLa cells by magnetic fractionation after “pulse chase” loading of dextran-coated iron-oxide nanoparticles (**Fig E in S1 Text**). PLD3 was highly enriched in the lysosomal fraction and accounted for the vast majority of the PLD3 in the cells (**Fig 2B**), confirming that PLD3 is primarily a lysosomal protein.

### PLD3 has phospholipase D activity in intact lysosomes

PLD3 is detected as two bands on western blot; the lower is reportedly derived from proteolytic cleavage of the transmembrane domain.[6] Consistent with this report, we found the higher molecular weight band segregated to the membrane fraction of human brain lysates (**Fig F in S1 Text**). We could detect no phospholipase D activity above baseline in total lysate of PLD3 transfected mammalian cells (**Fig 2A**). We suspected this was due to PLD3 constituting a small fraction of the total cellular PLD activity, as PLD1 and PLD2 are present as well. Consequently, we isolated lysosomes from PLD3-transfected NSC34 cells (a murine neuroblastoma line) to assess PLD activity, we found that PLD3 containing lysosomes had significantly increased phospholipase D activity compared to non-transfected lysosomes (**Fig 2D**). Importantly, overexpressed PLD3 continued to localize to the lysosome (**Fig B in S1 Text**). The specificity of the increase in PLD activity was evaluated by co-transfecting a validated siRNA against hPLD3 (validated in **Fig A in S1 Text**). Co-transfection with anti-PLD3 siRNA reduced the phospholipase D activity to that of lysosomes transfected with a scrambled siRNA (**Fig 2D**). The specificity of PLD3 activity via siRNA knockdown was replicated in a human cell line (**Fig G in S1 Text**). To further confirm that the phospholipase D activity originated from the conserved HKD domain, we introduced a mutation in the second HKD domain, K418R, which completely ablated the enzymatic activity of PLD3 (**Fig 2D,** source data in **S1 Data**). A plasmid containing a K203R mutation in the first HKD domain did not efficiently express full length modified protein. Finally, we found the human AD-associated V232M variant lacked PLD activity (**Fig 2D**). PLD3-V232M and PLD3-K203R continued to be trafficked to lysosomes (**Fig 2C**). Additionally, PLD3 transfection did not alter PLD1 or PLD2 levels in the lysosome (**Fig B in S1 Text**), which are known to be functional PLD enzymes. These data suggest PLD3 is a functional phosopholipase D in lysosomes.

### Variance in human PLD3 mRNA levels correlated with β-amyloid deposition and cognition in aged and AD participants

We evaluated whether stratifications in human PLD3 gene expression in the brain correlate with AD risk. The Religious Orders Study (ROS) and Rush Memory and Aging Project (MAP) enrolled older adults without dementia who agreed to annual clinical evaluations and brain donation [17–19]. A total of 531 ROS/MAP participants had mRNA expression levels measured from frozen sections of dorsolateral prefrontal cortex, as previously described [21], along with autopsy measures of neuropathology and premorbid longitudinal neuropsychological test performance. Participants were well educated, predominantly female, predominantly non-Hispanic white, and on average 89 (±7) years of age at death.

**Fig 3:**
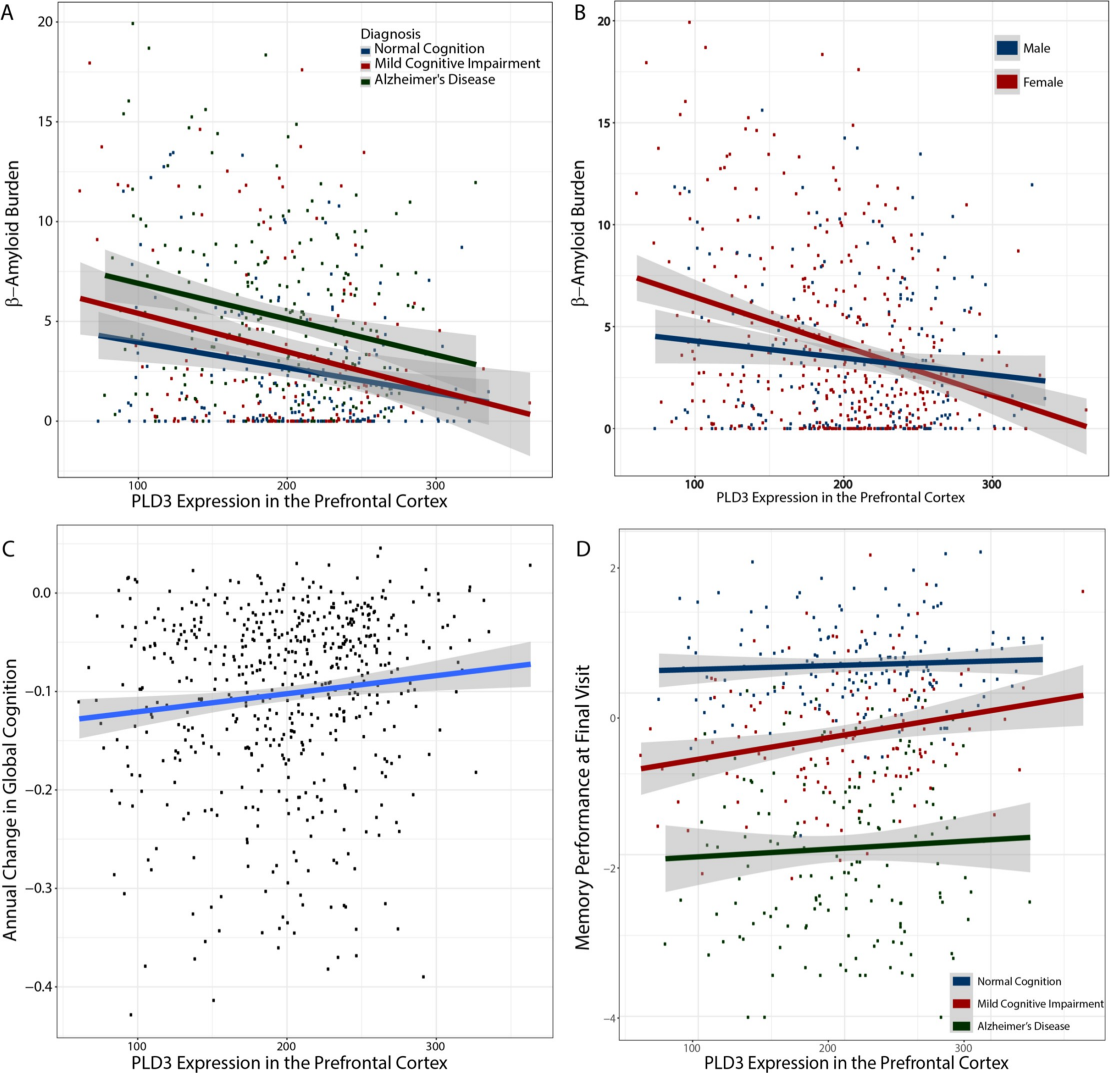
PLD3 expression correlates with neuropathology and cognitive trajectory. Human hippocampal PLD3 mRNA counts (in transcripts per million, TPM) were obtained from the ROS/MAP cohort **A.** Regression analysis reveals across all clinical diagnoses, higher levels of PLD3 appear protective with respect to lower β-amyloid plaque counts in prefrontal cortex. **B.** Protective effect of PLD3 expression on β-amyloid burden is stronger in women. **C.** Across all clinical diagnoses, higher levels of PLD3 correlate with slower rates of cognitive decline. **D.** PLD3 expression correlates with memory function.

In linear regression models covarying for age at death, sex, and postmortem interval, higher prefrontal cortex expression of *PLD3* was associated with lower β-amyloid plaque burden measured via immunolabeling, explaining 3% of the variance beyond covariates (**Fig 3A**; PLD3: β = -0.004, p = 1.7x10^-6^). β-amyloid plaque burden remained significantly associated when covarying for clinical diagnosis (p = 1.6x10^-5^). Similarly, higher prefrontal cortex expression of *PLD3* was associated with neurofibrillary tangle burden quantified by silver stain (β = -0.0006: p = 0.03) and IHC (β = -0.002, p = 0.03), however in both tangle models, inclusion of *in vivo* clinical diagnosis attenuated *PLD3* associations (p>0.07). When covarying for age at death, sex, and pos t mortem interval, lower levels of *PLD3* were observed among AD cases compared to cognitively normal participants (β = -11.9, p = 0.03). PLD3 levels were not associated with cerebral atherosclerosis (**Fig H in S1 Text**). In cognitive analyses, high prefrontal cortex expression of *PLD3* was associated with a slower rate of global cognitive decline when adjusting for age at death, sex, postmortem interval, and the interval between the final neuropsychological assessment and death (β = 0.0002, p = 0.02) (**Fig 3D**).

We observed a subtle sex difference in *PLD3* associations. Prefrontal cortex levels of *PLD3* interacted with sex on β-amyloid plaque density measured with IHC (β = 0.004, p = 0.02) (**Fig 3B**) and on cross-sectional global cognitive performance at the final visit before death (β = -0.004, p = 0.03). In both cases, *PLD3* associations were stronger in female than male participants. When performing sensitivity analyses for cell type fraction leveraging the abundance of cell-specific single gene markers (e.g., ENO2 for neurons, GFAP for astrocytes, CD68 for microglia), PLD3 associations with β-amyloid and cognitive decline remained significant (p<0.05).

### PLD3 mRNA levels are lower in AD-BXD mice and correlated with learning and memory performance

**Fig 4:**
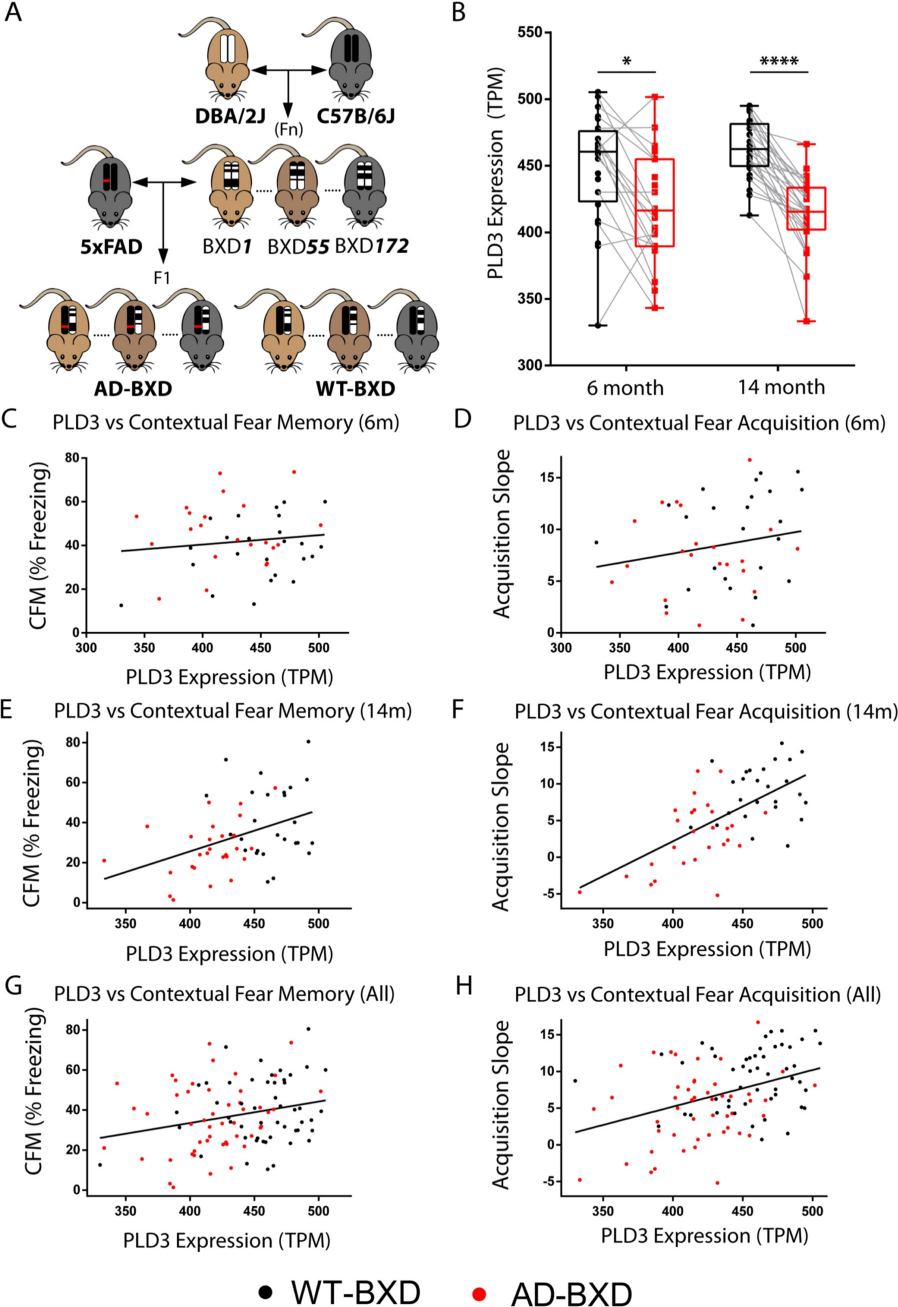
Variation in PLD3 expression predicts cognition in mice. **A-H.** The AD-BXD mouse model mimics human genetic diversity with respect to variation of PLD3 expression and subsequent learning and memory deficits. Mice underwent contextual fear testing (n = 636), of which a subset was euthanized and brain tissue subjected to RNA-Seq (n = 133). Dots represent averaged behavioral performance within BXD background strain and PLD3 transcript count. **A.** Schematic of approach to breeding AD-BXD mice; the AD mouse model 5xFAD (hemizygous) was crossed with genetically diverse strains from the BXD model [13]. Littermate controls were generated via this F1 cross due to the hemizygosity of the 5xFAD transgene. **B.** PLD3 hippocampal mRNA expression (displayed in normalized transcripts per million (TPM)) is widely stratified across BXD background and is reduced in AD-BXD mice at both 6 and 14 months (two-way ANOVA, *p<0.05 & ****p<0.0001 following Bonferroni post-hoc tests). Gray connecting lines indicate shared sex and BXD background strain for visual comparison. **C-H.** Contextual fear conditioning behavioral performance regarding fear memory recall (**C, E, G**) and fear learning acquisition speed (**D, F, H**) plotted against the group averaged PLD3 mRNA transcript count. Pooled cohort of mice display positive correlation with hippocampal PLD3 mRNA expression and fear memory recall (**G**) and fear learning acquisition speed (**H**), with the correlational slope more strongly affected by age in memory recall (**E vs C**) and acquisition speed (**F vs D**).

The BXD mouse model was created by crossing a wild-type mouse with C57BL/6J background with a DBA/2J mouse to produce a family of inbred sub-strains that capture a range of genetic diversity. AD-BXD mice were produced by crossing the resulting BXD strains with 5xFAD hemizygotes, an animal model containing five pro-β-amyloidosis mutations [14] (**Fig 4A**). PLD3 mRNA levels were significantly lower in AD-BXD mice than their wild type BXD counterparts (**Fig 4B**). Mice were subjected to a modified contextual fear memory test (as previously described [13]) due to its well-validated sensitivity to discern learning and memory impairments in multiple AD mouse models [37,38]. On the first experimental day, freezing behavior was measured following repeated mild foot-shocks to assess learning acquisition rates and the second day freezing behavior in the same environmental context without the presentation of foot-shocks was measured for analysis of memory recall. Following behavioral experimentation at the respective time points, a WT and 5xFAD mouse from each strain was euthanized to analyze mRNA levels in hippocampus via RNA-Seq. We modeled strain-averaged behavioral performance against PLD3 mRNA transcript counts and found that PLD3 levels (adjusting for age and sex) predicted learning acquisition slope (β = 0.06, p = 2.8x10^-6^, explaining 6% of the variance) (**Fig 4D, 4F and 4H**). When adjusting for age and sex, PLD3 levels were a significant predictor of fear memory recall (β = 0.09, p = 0.03) (**Fig 4C, 4E, and 4G**). When including genotype and BXD background strain, PLD3 remained predictive of learning acquisition speed (β = 0.04, p = 0.003) but not when modeling fear memory recall (β = 0.085, p = 0.08). These findings are unsurprising as the main effect for the AD-BXD behavioral stratification was observed upon learning acquisition slope rather than memory recall, as recall is more strongly sensitive to β-amyloid burden [14,15]. Collectively, these human and animal data suggest that PLD3 expression is linked to cognition in the aged brain. Of note pertaining to the reports of motor deficits with PLD3 hypofunction [6], PLD3 levels were not predictive of narrow beam balance in the BxD cohort (**Fig I in S1 Text**).

## Discussion

Rare PLD3 coding gene variants conferring an increased risk of late onset AD were discovered in 2014 but have remained controversial in large part due to the unknown function of PLD3 and unclear molecular impact of the rare variant [1]. Here, we leveraged multimodal datasets that reflect the role of PLD3 at a population level in both a large, longitudinal human study and in a genetically diverse murine model to establish a functional connection between PLD3 expression and AD-related cognitive impairment. In humans, we found a protective association between increased PLD3 expression and β-amyloid plaque burden. Increased PLD3 expression correlated with reduced rate of cognitive decline in the ROSMAP cohort (**Fig 3**) and learning and memory in the AD-BXD mice (**Fig 4**). While the minor allele frequency for the AD-associated PLD3 variant is only around 1% [39], these results advance our understanding of the functional connection between PLD3 expression and cognition to establish that PLD3 may be relevant to AD at a population level.

Endolysosomal dysfunction has become an important research focus in age-related neurodegenerative diseases, as many have been linked to lysosomal genes, including, for example, progranulin mutations in frontotemporal dementia and glucocerebrocidase 1 mutations in Parkinson’s disease.[40,41] In AD, dystrophic neurites are a consistent pathological feature of AD made up of enlarged axon segments around β-amyloid plaques clogged with dysfunctional lysosomes, but unlike the prior examples where haploinsufficiency of key genes accounts for the lysosomal defect, the mechanism(s) of lysosomal function in AD are less clear at present [36,42]. Here, we report PLD3 is highly concentrated in abnormal neuronal lysosomes in axons surrounding β-amyloid plaques (**Fig 1**), corroborating earlier reports of PLD3 upregulation around β-amyloid plaques [43] and that PLD3 can be routed to the lysosome [34]. The close and consistent association of PLD3 with β-amyloid neuropathology further strengthens the relevance of PLD3 to AD.

Finally, we evaluated the molecular function of PLD3. Because PLD3 is lysosomal at endogenous expression levels, we hypothesized that its activity likely requires an acidic pH or some other feature(s) of the lysosomal microenvironment. Consistent with initial reports [6,7], we did not detect a change in phospholipase D activity in total cell lysates after overexpressing PLD3, which is not surprising given lysosomes account for well under 5% of the total lysate and several other PLD isoforms are present in the lysate. However, when we isolated lysosomes from transfected mammalian cell lines, we discovered markedly enhanced PLD activity after transfecting PLD3, which was ablated when an anti-PLD3 siRNA was co-transfected or a mutation in the putative active site was introduced. Using this assay, we demonstrated that the AD-associated PLD3 variant, V232M, lacked PLD activity. This discovery strengthens the case that PLD3-V232M is a bonafide AD risk gene. Moreover, these results give us confidence that PLD3 is a mammalian PLD isoform.

An alternative molecular function of PLD3 has been proposed. A recent report argued that both PLD3 and PLD4 have 5’ exonuclease activity and implicated this activity in the function of antigen presenting cells in the context of autoimmune diseases [3]. PLD4 variants have been linked to autoimmune disease, particularly rheumatoid arthritis [44–46], but there is no such association with PLD3. Moreover, we found PLD3 was nearly exclusively expressed in neurons in the brain and was essentially absent from microglia. PLD3 knock-out mice have been reported to have increased lysosomal size in neurons [8], and an alteration in neuronal lysosomal membrane dynamics (fission or fusion) is more consistent with PLD activity than with exonuclease activity. It may ultimately be necessary to decipher the structure of PLD3 in greater detail than currently available to confirm the primary molecular function. Importantly, however, whether PLD3 is a PLD or 5’ exonuclease, the HKD domains appear to be enzymatically active which raises the probability that an inactivating mutation in PLD3 is truly pathogenic. Both PLD1 and PLD2 require various activating factors for *in vitro* activity (including protein kinase C, PIP2 or ARL1) [47,48], it is therefore highly likely that PLD3 requires similar factors which are currently unknown but are presumably present in the lysosomal microenvironment.

While it is possible that PLD3 enrichment in dystrophic neurites represents a compensatory cellular response to plaque formation, we hypothesize that PLD3 is functionally inactive in these sites. While considerable protease immunoreactivity has been observed around β-amyloid plaques, lysosomes in dystrophic neurites appear to be functioning sub-optimally based on our observation that they are deficient in lysosomal proteases [35,38,49] compared to lysosomes in neuronal cell bodies (**Fig 1 and Fig B in S1 Text**). Given this functional defect, it is reasonable to hypothesize that they might also fail to acidify, and if PLD3 required acidic pH to be active, it could be functionally inactive in dystrophic neurites. This highlights the need for further detailed studies to define the physiology of lysosomes within dystrophic neurites. It may be possible to interrogate dystrophic neurites *ex vivo* via organotypic tissue culture [50] using tissue from the 5xFAD mouse or other AD mouse models. Together, these data warrant further investigation towards the potential protective effect of enhancing PLD3 activity to disrupt AD pathogenesis.

## Supporting information

S1 Text **Table A:** Neuropathology subjects’ clinical characteristics. Human AD Brain Tissue Utilized for Neuropathological Immunohistochemistry. **Table B:** Antibodies Utilized and Concentrations Used. **Fig A:** Endogenous PLD3 was detected in HeLa cells as a pair of bands on western blot at 55 and 65 kDa. These bands were eliminated by transfection of an siRNA against the human PLD3 transcript and amplified many fold by transfection of a plasmid containing an untagged PLD3 transcript. Manipulating the level of PLD3 did not alter the level of APP, confirming a previous report [7]. **Fig B:** PLD3 transfection does not alter lysosomal level of PLD1 or PLD2. **Fig C:** PLD3 is associated with lysosomes in human brain and massively enriched on abnormal lysosomes in dystrophic neurites. PLD3 closely colocalized with neuronal lysosomes and co-labeled with cathepsin D which is a luminal lysosomal protease, as well as LAMP2 and progranulin with are components of the lysosomal membrane. PLD3 accumulations around β-amyloid plaques co-label with LAMP2 and progranulin. Lysosomes in dystrophic neurites are deficient in cathepsins as has been previously reported in animal models of Alzheimer’s disease. **Fig D:** Staining human brain axons (neurofilament in red), β-amyloid (methoxy-XO4 in blue) and PLD3 (in green) demonstrates that PLD3 is not associated with cerebral amyloid angiopathy in arterioles. However, it is robustly present in dystrophic neurites around capillaries with cerebral amyloid angiopathy. **Fig E:** A. HeLa cells stably transfected with GFP-tagged transcription factor EB (TFEB), a transcription factor central to the initiation of lysosomal biogenesis, demonstrate cytosolic localization of TFEB in basal conditions. Upon induction of lysosomal biogenesis, TFEB rapidly translocates to the nucleus. To ensure that loading of dextran-coated small paramagnetic iron oxide nanoparticles (SPIO) did not induce lysosomal biogenesis, we demonstrated that 12-hour incubation with the nanoparticles did not induce TFEB translocation. B. Efficiency and purity of lysosome enrichment of lysosome isolate by magnetic chromatography are demonstrated by the enrichment of lysosome markers LAMP1, LAMP2 and Cathepsin D in the lysosomal fraction, without enrichment of mitochondrial marker manganese superoxide dismutase (Mn SOD). **Fig F:** PLD3 in human superior temporal gyrus. The higher molecular weight band is detected in the membrane fraction while the lower molecular weight band is primarily in the soluble fraction. This is consistent with prior reports that the lower molecular weight PLD3 band is derived from cleavage of the transmembrane domain. LAMP2 is associated with the lysosomal membrane while Cathepsin D is present in both fractions as expected. To isolate the soluble fraction, the supernatant was collected after tissue was homogenized in three volumes of TBS with phosphatase and protease inhibitors and centrifuged at 100,000g for one hour. The membrane fraction was obtained by resuspending the pellet in three volumes of TBS with 1% Triton X, rotating end-over-end for 1 hour, the centrifuging at 100,000g for one hour. The resulting supernatant contained the membrane fraction. **Fig G:** Replication study in HeLa cells reveals that PLD3 has enzymatic PLD activity in human cell line derived lysosomes. Lysosomes isolated from PLD3 transfected HeLa cells display significantly increased PLD activity compared to control lysosomes (p<0.0001). Cotransfection of PLD3 and siRNA against PLD3 significantly reduces lysosomal PLD activity (p<0.0001), indicating the specificity of the enzymatic activity of PLD3. PLD activity in PLD3+siRNA compared to control is not significant (p = 0.48). **Fig H:** PLD3 mRNA levels do not correlate with cerebral atherosclerotic disease. **Fig I:** PLD3 mRNA levels in 5x/BXD and wild-type BXD mice do not correlate with narrow beam performance. Linear regression reveals the plotted slope is not statistically non-zero (p>0.1), and this does not change upon covarying for 5xFAD genotype (p = 0.475). TPM = transcripts per million, y-axis in seconds.
(DOCX)

S1 Data Excel file containing source data for PLD activity in Fig 2. (XLSX)
