# Supplementary material for: PLD3 is a neuronal lysosomal phospholipase D associated with β-amyloid plaques and cognitive function in Alzheimer’s disease
Source: PLoS Genet. 2021 Apr 8;17(4):e1009406. doi: 10.1371/journal.pgen.1009406 (PMC8031396; doi:10.1371/journal.pgen.1009406)

**Supplemental Materials**

**Supplemental Table A**. Neuropathology subjects’ clinical characteristics. Human AD Brain Tissue Utilized for Neuropathological Immunohistochemistry

**Supplemental Table B.** Antibodies Utilized and Concentrations Used

**Supplemental Figure A.** Endogenous PLD3 was detected in HeLa cells as a pair of bands on western blot at 55 and 65 kDa. These bands were eliminated by transfection of an siRNA against the human PLD3 transcript and amplified many fold by transfection of a plasmid containing an untagged PLD3 transcript. Manipulating the level of PLD3 did not alter the level of APP.

**Supplemental Figure B.** PLD3 transfection does not alter lysosomal level of PLD1 or PLD2

**Supplemental Figure C.** PLD3 is associated with lysosomes in human brain and massively enriched on abnormal lysosomes in dystrophic neurites. PLD3 closely colocalized with neuronal lysosomes and co-labeled with cathepsin D which is a luminal lysosomal protease, as well as LAMP2 and progranulin with are components of the lysosomal membrane. PLD3 accumulations around β-amyloid plaques co-label with LAMP2 and progranulin. Lysosomes in dystrophic neurites are deficient in cathepsins as has been previously reported in animal models of Alzheimer’s disease.

**Supplemental Figure D:** Staining human brain axons (neurofilament in red), β-amyloid (methoxy-XO4 in blue) and PLD3 (in green) demonstrates that PLD3 is not associated with cerebral amyloid angiopathy in arterioles. However, it is robustly present in dystrophic neurites around capillaries with cerebral amyloid angiopathy.

**Supplemental Figure E.** A. HeLa cells stably transfected with GFP-tagged transcription factor EB (TFEB), a transcription factor central to the initiation of lysosomal biogenesis, demonstrate cytosolic localization of TFEB in basal conditions. Upon induction of lysosomal biogenesis, TFEB rapidly translocates to the nucleus. To ensure that loading of dextran-coated small paramagnetic iron oxide nanoparticles (SPIO) did not induce lysosomal biogenesis, we demonstrated that 12-hour incubation with the nanoparticles did not induce TFEB translocation. B. Efficiency and purity of lysosome enrichment of lysosome isolate by magnetic chromatography are demonstrated by the enrichment of lysosome markers LAMP1, LAMP2 and Cathepsin D in the lysosomal fraction, without enrichment of mitochondrial marker manganese superoxide dismutase (Mn SOD).

**Supplemental Figure F.** PLD3 in human superior temporal gyrus. The higher molecular weight band is detected in the membrane fraction while the lower molecular weight band is primarily in the soluble fraction. This is consistent with prior reports that the lower molecular weight PLD3 band is derived from cleavage of the transmembrane domain. LAMP2 is associated with the lysosomal membrane while Cathepsin D is present in both fractions as expected. To isolate the soluble fraction, the supernatant was collected after tissue was homogenized in three volumes of TBS with phosphatase and protease inhibitors and centrifuged at 100,000g for one hour. The membrane fraction was obtained by resuspending the pellet in three volumes of TBS with 1% Triton X, rotating end-over-end for 1 hour, the centrifuging at 100,000g for one hour. The resulting supernatant contained the membrane fraction.

**Supplemental Figure G.** Replication study in HeLa cells reveals that PLD3 has enzymatic PLD activity in human cell line derived lysosomes. Lysosomes isolated from PLD3 transfected HeLa cells display significantly increased PLD activity compared to control lysosomes (p<0.0001). Cotransfection of PLD3 and siRNA against PLD3 significantly reduces lysosomal PLD activity (p<0.0001), indicating the specificity of the enzymatic activity of PLD3. PLD activity in PLD3+siRNA compared to control is not significant (p=0.48).

**Supplemental Figure H.** PLD3 mRNA levels do not correlate with cerebral atherosclerotic disease.

**Supplemental Table A.** Antibodies utilized and concentrations used

|  | Concentration | | |  |  |
| --- | --- | --- | --- | --- | --- |
| Antibody | WB | hIHC | mIHC | Supplier | Product ID |
| PLD1 | 1:50 | - | - | Cell Signaling | 3832 |
| PLD2 | 1:500 | - | - | Cell Signaling | 13904 |
| PLD3 | 1:250 | 1:100 | 1:100 | Sigma | HPA012800 |
| LAMP1 | 1:500 | - | - | DSHB | 1DB4-S |
| LAMP2 | 1:500 | 1:250 | 1:250 | Santa Cruz | sc-18822 |
| Cathepsin B | - | 1:200 | 1:200 | R&D | AF965 |
| Cathepsin D | 1:500 | 1:200 | - | R&D | AF1014 |
| APP | 1:500 | - | - | Biolegend | 802801 |
| Tubulin | 1:500 | - | - | Santa Cruz | sc-8035 |
| Progranulin | - | 1:200 | - | R&D | AF2557 |

WB: Western Blot, hIHC: human immunohistochemistry, mIHC: mouse immunohistochemistry

**Supplemental Table B**. Neuropathology subjects’ clinical characteristics

| **Subject** | **Age/Sex** | **Braak and Braak grade, duration** | **Cerebral amyloid angiopathy** | **Cause of death** | **Family history of dementia** | **Major comorbidities** |
| --- | --- | --- | --- | --- | --- | --- |
| AD1 | 64 / M | VI, 7 years | Mild | Pneumonia | No (PS1 -) | None |
| AD2 | 79 / M | VI, 10 years | None | Stroke | No | Diabetes, papillary thyroid carcinoma, atherosclerosis |
| AD3 | 81 / F | VI, 7 years | Mild | Pneumonia | 2 siblings | Atrial fibrillation |
| AD4 | 82 / F | IV, 5 years | None | Pneumonia | No | None |
| AD5 | 93 / M | III, 3 years | Severe | Pneumonia | No | None |
| AD6 | 62 / M | I, none | Severe | Intracerebral hemorrhage | No | None |
| AD7 | 86 / M | VI, unknown (longstanding) | Severe | Intracerebral hemorrhage | No | Hypertension |
| AD8 | 71 / M | VI, 7 years | Severe | Intracerebral hemorrhage | No | Waldenstrom’s macroglobulinemia, diabetes |
| Control 1 | 27 / M | None | None | Congenital heart disease | No | None |
| Control 2 | 29 / F | None | None | Arrhythmia | No | None |
| Control 3 | 45 / M | None | None | Basilar artery occlusion | No | Atherosclerosis, hypertension, diabetes |
| Control 4 | 57 / F | None | None | Sepsis | No | Perforated colon |
| Control 5 | 62 / M | None | None | Sepsis | No | C.dificil colitis, liver transplant recipient |
| Control 6 | 69 / F | None | None | Heart failure | No | Dilated cardiomyopathy |
| Control 7 | 77 / M | None | None | Sepsis | No | Lymphoma, neutropenia, atrial fibrillation. |
| Control 8 | 80 / F | None | Mild | Ischemic heart disease | No | Atherosclerosis, neuroendocrine lung tumor |

**Supplemental Figure A**. Anti-PLD3 siRNA depletes PLD3 levels


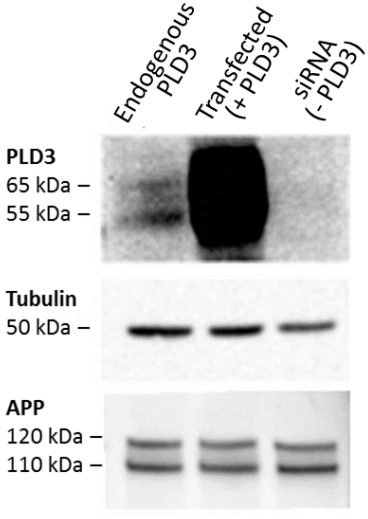


**Supplemental Figure B**. PLD3 transfection does not alter lysosomal level of PLD1 or PLD2


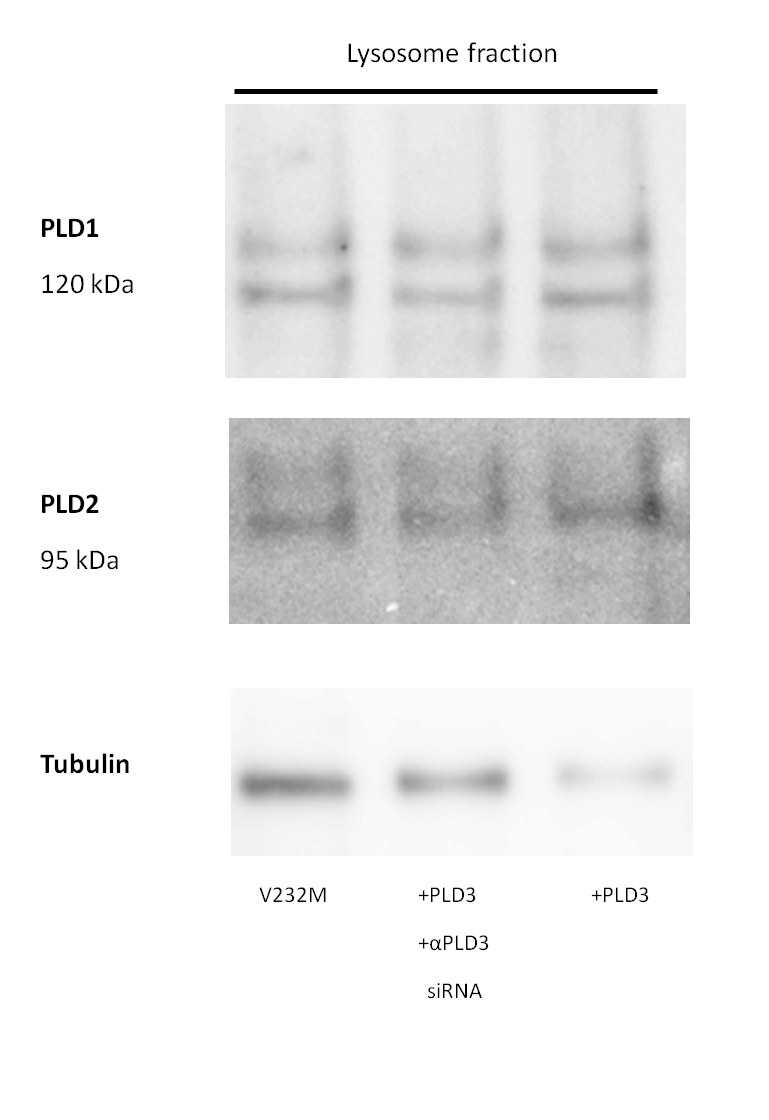


**Supplemental Figure C**. PLD3 colocalizes with numerous markers of lysosomes in human brain


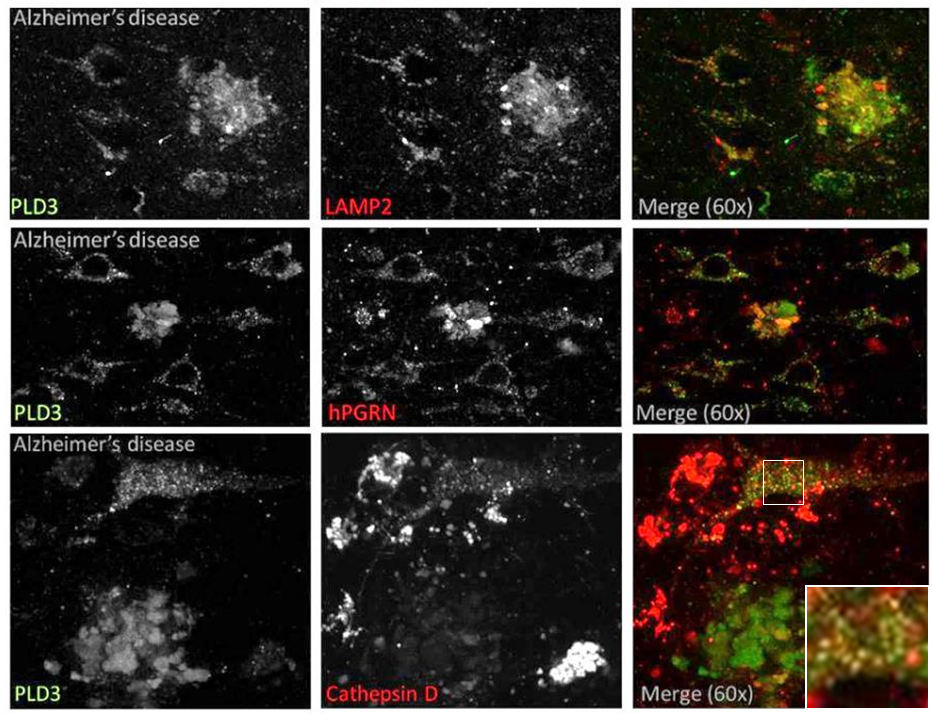


**Supplemental Figure D.**


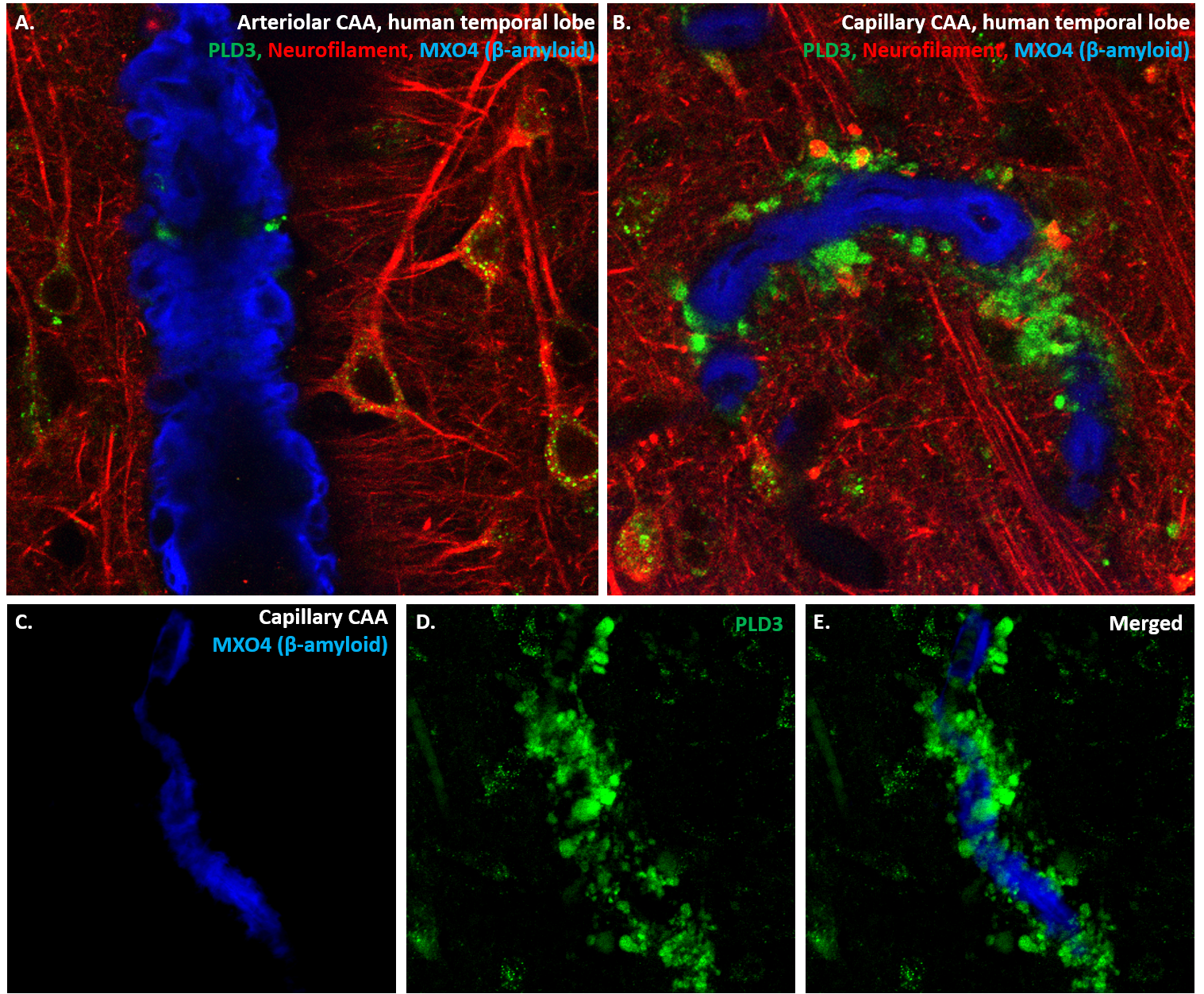


**Supplemental Figure E**. Lysosome purification procedure validation


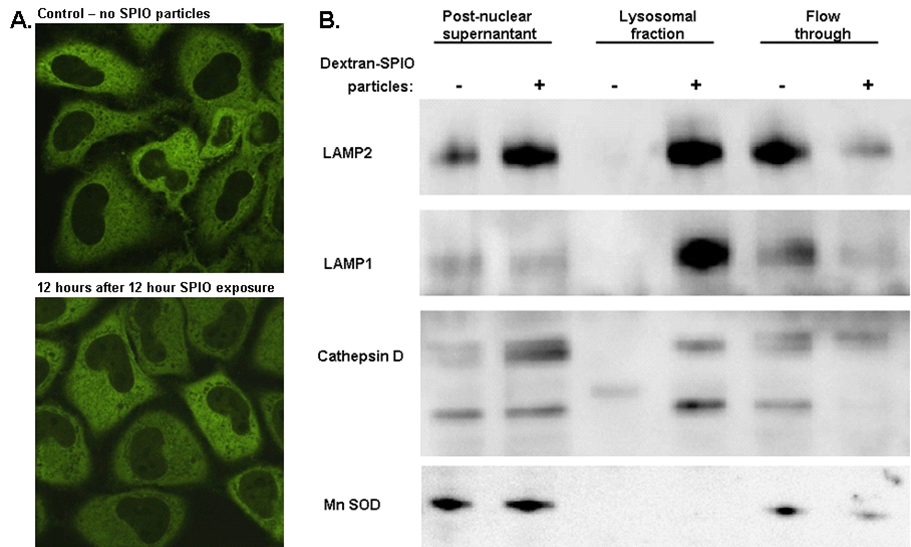


**Supplemental Figure F.**

**
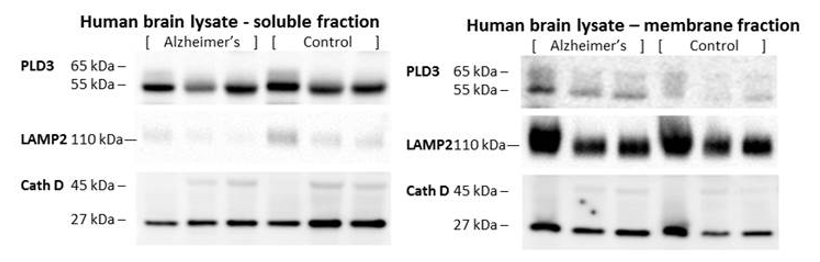
**

**Supplemental Figure G**. HeLa derived transfected lysosomes replicate PLD3 specific enzyme function


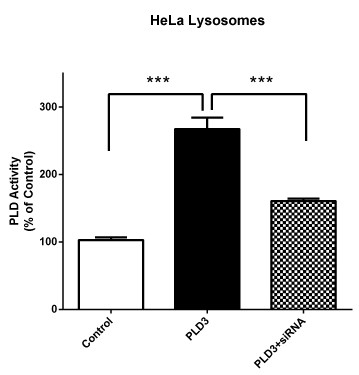


**Supplemental Figure H**. PLD3 mRNA levels do not correlate with cerebral atherosclerotic disease


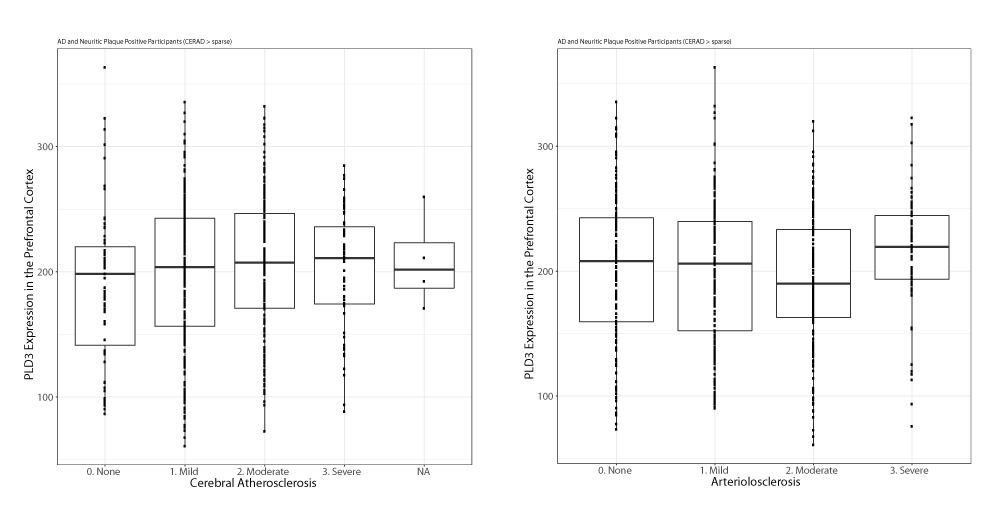

Supplement: S1 Text — Table A: Neuropathology subjects’ clinical characteristics. Human AD Brain Tissue Utilized for Neuropathological Immunohistochemistry. Table B: Antibodies Utilized and Concentrations Used. Fig A: Endogenous PLD3 was detected in HeLa cells as a pair of bands on western blot at 55 and 65 kDa. These bands were eliminated by transfection of an siRNA against the human PLD3 transcript and amplified many fold by transfection of a plasmid containing an untagged PLD3 transcript. Manipulating the level of PLD3 did not alter the level of APP, confirming a previous report [7]. Fig B: PLD3 transfection does not alter lysosomal level of PLD1 or PLD2. Fig C: PLD3 is associated with lysosomes in human brain and massively enriched on abnormal lysosomes in dystrophic neurites. PLD3 closely colocalized with neuronal lysosomes and co-labeled with cathepsin D which is a luminal lysosomal protease, as well as LAMP2 and progranulin with are components of the lysosomal membrane. PLD3 accumulations around β-amyloid plaques co-label with LAMP2 and progranulin. Lysosomes in dystrophic neurites are deficient in cathepsins as has been previously reported in animal models of Alzheimer’s disease. Fig D: Staining human brain axons (neurofilament in red), β-amyloid (methoxy-XO4 in blue) and PLD3 (in green) demonstrates that PLD3 is not associated with cerebral amyloid angiopathy in arterioles. However, it is robustly present in dystrophic neurites around capillaries with cerebral amyloid angiopathy. Fig E: A. HeLa cells stably transfected with GFP-tagged transcription factor EB (TFEB), a transcription factor central to the initiation of lysosomal biogenesis, demonstrate cytosolic localization of TFEB in basal conditions. Upon induction of lysosomal biogenesis, TFEB rapidly translocates to the nucleus. To ensure that loading of dextran-coated small paramagnetic iron oxide nanoparticles (SPIO) did not induce lysosomal biogenesis, we demonstrated that 12-hour incubation with the nanoparticles did not [file pgen.1009406.s001.docx]
